# Surgical treatment of skeletal metastases in proximal tibia: a multicenter case series of 74 patients

**DOI:** 10.1080/17453674.2020.1866242

**Published:** 2021-01-07

**Authors:** Kaarel Kilk, Jessica Ehne, Jonathan D Stevenson, Gilber Kask, Jyrki Nieminen, Rikard Wedin, Michael C Parry, Minna K Laitinen

**Affiliations:** a Department of Orthopaedics, Helsinki University Hospital and University of Helsinki, Helsinki, Finland;; b Department of Orthopaedics, Tampere University Hospital, Tampere, Finland;; c Department of Reconstructive Orthopaedics, Karolinska University Hospital, Stockholm, Sweden;; d Department of Orthopaedics, Royal Orthopaedic Hospital, Birmingham, UK;; e Aston University Medical School, Aston University, Birmingham, UK;; f Coxa, Hospital for Joint Replacement, Tampere, Finland

## Abstract

Background and purpose — The proximal tibia is a rare site for metastatic bone disease and is a challenging anatomical site to manage due to the proximity to the knee joint and poor soft tissue envelope. We investigated implant survival and complications of different surgical strategies in the treatment of proximal tibia pathological fractures.

Patients and methods — The study comprised a 4 medical center, retrospective analysis of 74 patients surgically treated for metastases of the proximal tibia. Patient records were reviewed to identify outcome, incidence, and type of complications as well as contributing factors.

Results — Reconstruction techniques comprised cement-augmented osteosynthesis (n = 33), tumor prosthesis (n = 31), and total knee arthroplasty with long cemented stems (n = 10). Overall implant survival was 88% at 6 months and 1 year, and 67% at 3 years. After stratification by technique, the implant survival was 82% and 71% at 1 and 3 years with tumor prosthesis, 100% at 1 and 3 years with total knee arthroplasty, and 91% at 1 year and 47% at 3 years with osteosynthesis. Preoperative radiotherapy decreased implant survival. Complications were observed in 19/74 patients. Treatment complications led to amputation in 5 patients.

Interpretation — In this study, the best results were seen with both types of prothesis reconstructions, with good implant survival, when compared with treatment with osteosynthesis. However, patients treated with tumor prosthesis showed an increased incidence of postoperative infection, which resulted in poor implant survival. Osteosynthesis with cement is a good alternative for patients with short expected survival whereas endoprosthetic replacement achieved good medium-term results.

The most common site for skeletal metastases requiring surgical intervention is the proximal femur, which accounts for approximately 65% of all cases. In contrast, the tibia accounts for only 3% of pathological fractures requiring surgery, mostly commonly in the proximal third (Ratasvuori et al. 2013). Due to the proximity of the knee joint and the poor soft tissue envelope in the proximal tibia, the management of metastatic deposits and pathological fractures in this region can be challenging.

Given the scarcity of this metastasis location, there is a paucity of evidence relating to the outcomes of surgical treatment of pathological fractures of the proximal tibia. In this retrospective case series we assessed the advantages and disadvantages of different surgical reconstructions, looking in particular at implant survival, the incidence of complications, and the possible factors that may affect these outcomes.

## Patients and methods

The study comprised a retrospective analysis of all patients treated for a complete or pending pathological fracture arising within the proximal tibia treated at 1 of 4 international collaborative hospitals: Helsinki University Hospital, Helsinki, Finland;, Coxa Hospital for Joint replacement, Tampere, Finland; Karolinska University Hospital, Stockholm, Sweden; and Royal Orthopaedic Hospital, Birmingham, UK. The study population comprised 74 patients treated between 2000 and 2018. All patients over 18 years of age with histologically confirmed metastatic bone disease of any primary malignancy, including multiple myeloma and lymphoma, were included. The decision to undergo surgical intervention was discussed at a multidisciplinary team conference at each of the 4 centres and was made following discussion between the operating surgeon and the patient. Preoperative radiological assessment comprised plain radiographs in all cases, and in selected cases with computed tomography (CT) or magnetic resonance imaging (MRI). Systemic staging comprised CT scan of the chest, abdomen, and pelvis, and whole-body skeletal imaging in the form of radiolabelled technicium bone scan.

**Figure 1.**
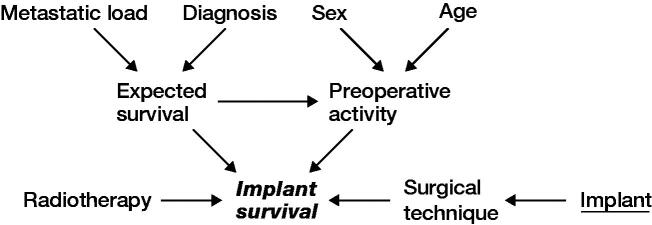
Causal pathways in directed acyclic graphs in the variable selection. Exposure of interest = implant survival, outcome = implant survival, suggested covariates, sex, age, diagnosis, metastatic load, radiotherapy.

Data was extracted from prospectively maintained institutional databases as well as medical records. Radiological assessment of relevant imaging was undertaken to assess eligibility. Patient- and reconstruction-related outcome measures were recorded. A causal-directed acyclic graph (DAG) was used to investigate confounding factors (Figure 1).

**Figure 2.**
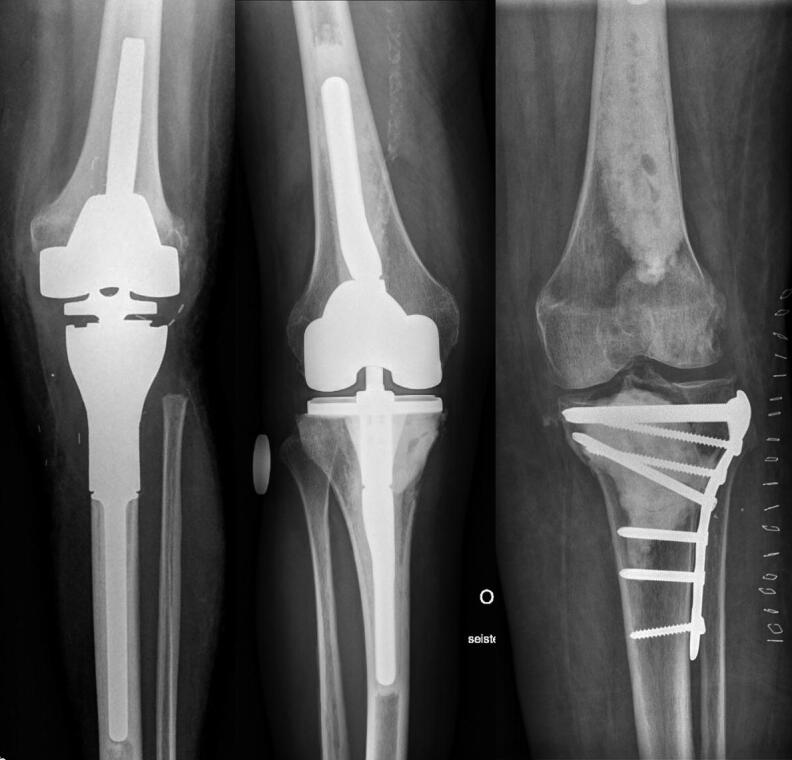
Surgical treatment methods: tumor prosthesis (A), total knee arthroplasty with long cemented stems (B), osteosynthesis using plate and cement (C).

The surgical treatment methods for impending or pathological fracture were stratified into 1 of 3 possible reconstructions: tumor prosthesis, total knee arthroplasty (TKA) with long cemented stems, or osteosynthesis using plate and cement (Figure 2).

Postoperative complications, including mechanical complications, were defined according to the classification by Henderson et al. (2014) (Table 1). Additionally, complications were defined as minor and major. Major complications were defined as those that required further surgical intervention. Minor complications were defined as those that did not require further surgical intervention.

**Table 1.** Patient characteristics (N = 74). Values are count unless otherwise specified

Male/Female	55/29
Impending fracture	64
Preoperative radiotherapy	12
Primary tumor	
Renal cell carcinoma	29
Melanoma	8
Colon carcinoma	6
Non-small-cell lung carcinoma	5
Sarcoma	5
Myeloma	5
Breast carcinoma	5
Prostate carcinoma	3
Esophagus	2
Bladder	2
Lymphoma	2
Retinoblastoma	1
Squamocellular	1
Median age, years ^a^	64 (18–86)
Mean follow-up, months ^a^	12 (0–210)
Mean size, cm^a^	6.3 (3–16)
Operative method	
Tumor prosthesis	31
Total knee arthroplasty with	
long cemented stems	10
Osteosynthesis and cement	33
Complications	19
Complications according to	
Henderson’s classification	18
Type 1	2
Type 2	0
Type 3	2
Type 4	8
Type 5	6
Revision surgery	13

**^a^** Range in parenthesis

### Statistics

Patient and implant survival rates were assessed using the Kaplan–Meier methods with 95% confidence intervals (CI). Between-group comparisons were performed using the log-rank test. Patient follow-up time was calculated from the date of surgery to the most recent follow-up date or the date of death. Implant survival was calculated from the date of surgery to revision surgery due to any cause. Continuous variables are reported as medians. The chi-squared test or Fisher’s exact test was used to compare variables between groups, and the Mann–Whitney U-test test for medians between groups. Subdistribution hazard ratio (SHR) of the role of factors affecting implant survival was calculated using competing risk analysis, where death was considered as a competing event. Statistical analyses were performed using SPSS Statistics 23.0 (IBM Corp, Armonk, NY, USA) but competing risk analysis was performed using STATA 16 (StataCorp, College Station, TX, USA). A p-value < 0.05 was considered significant.

### Ethics, funding, and potential conflicts of interest

This retrospective study was approved by the local chairs of the audit department. This research study received a grant from the state funding for university-level health research. The funder had no role in study design, data collection, and analysis, decision to publish, or preparation of the manuscript. No competing interests are declared.  

## Results

The study population comprised 74 patients (45 men) with a median age at the time of surgery of 64 years (18–86). The median follow-up period was 12 months (0–210) and at final follow-up, 26 patients were alive. The most common primary malignancy was renal cell carcinoma (RCC, n = 29), followed by melanoma (8), colon cancer (6), breast cancer, sarcoma, lung cancer, and myeloma (5 cases each). Of the 74 patients, 64 patients had an impending fracture and 10 patients had a complete fracture (Table 1). The overall mortality was 64% during the period of follow up. Overall patient survival after 6 months was 74% (61–83), at 1 year 58% (46–70), and at 3 years, 33% (21–45). Statistically significant factors negatively associated with survival were the incidence of a major complication (p = 0.04) and the presence of an actual pathological fracture (p = 0.02).

### Implant survival

**Figure 3.**
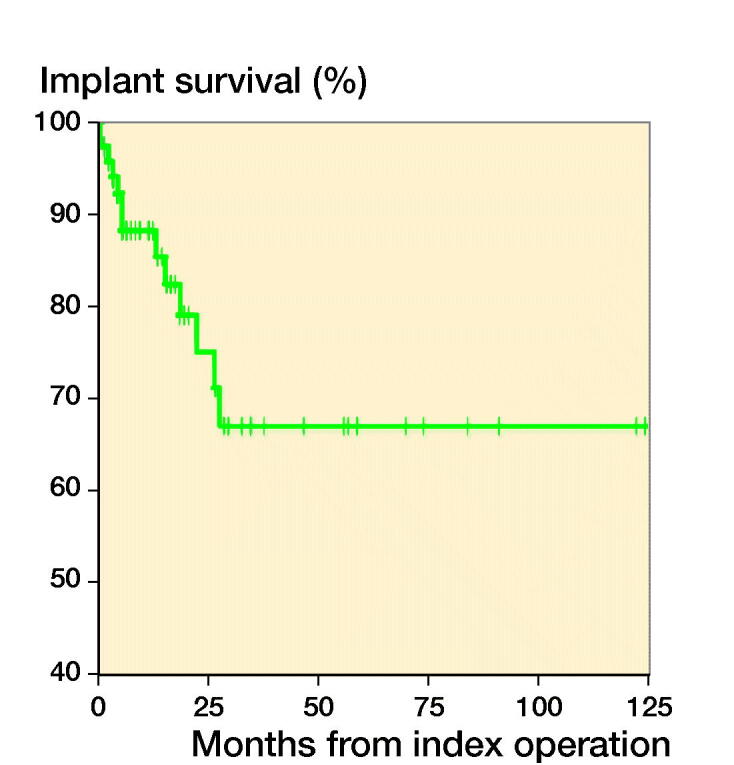
Implant survival.

**Figure 4.**
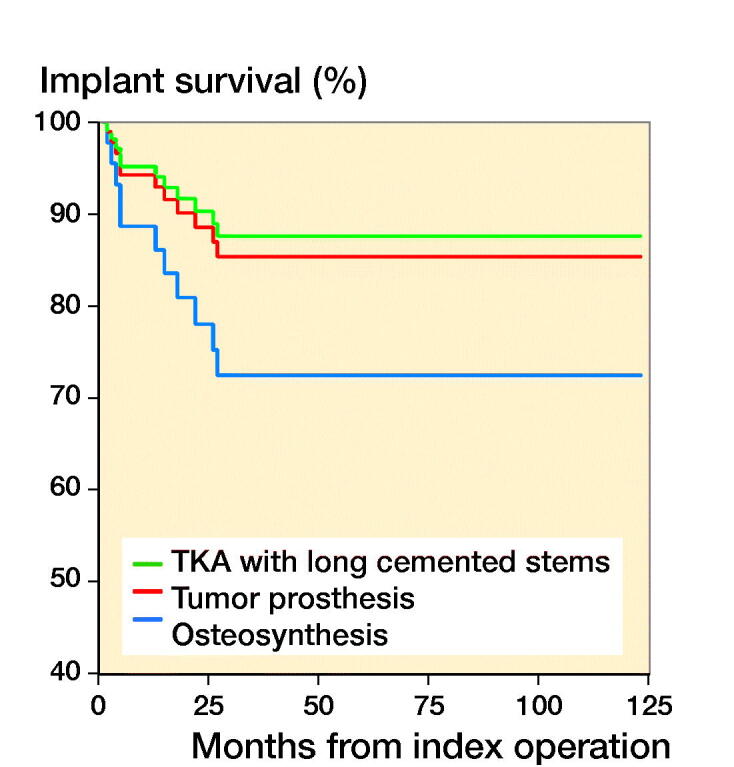
Implant survival stratified by surgical method in a competing risk model.

**Figure 5.**
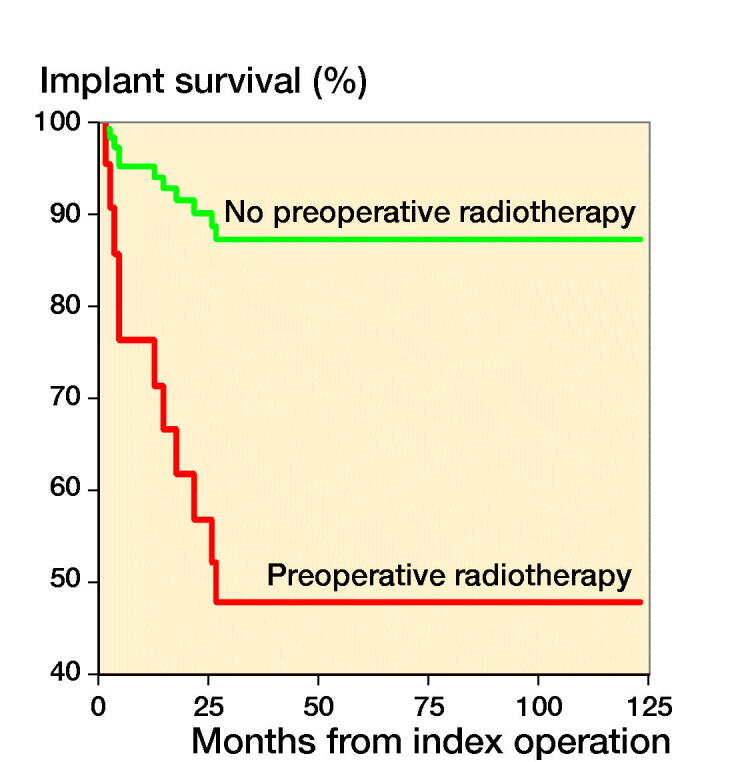
Implant survival stratified by radiotherapy in a competing risk model.

The chosen reconstruction method is given in Table 1. Overall implant survival, regardless of reconstruction technique, was 88% (80–97) after 6 months and 1 year, and 67% (50–83) at 3 and 5 years (Figure 3). After stratifying by operative method, implant survival for the tumor prosthesis group was 93% (84–100) at 6 months, 82% (67–96) at 1 year, and 71% (52–90) at 3 and 5 years. In the osteosynthesis group, the implant survival was 91% (79–100) at 6 months and 1 year, and 47% (15–79) at 3 and 5 years. In the total knee arthroplasty (TKA) with long cemented stem group, implant survival was 100% after 6 months, 1, 3, and 5 years. The effect of preoperative radiotherapy and reconstruction technique on implant survival was analysed using a competing risk model. There was worse implant survival for the osteosynthesis group, but without statistical significance (p = 0.2) (Figure 4). Preoperative radiotherapy had a significantly negative effect on implant survival compared with no preoperative radiotherapy in the tumor prosthesis and osteosynthesis groups (p = 0.004) (Figure 5). No other factors, identified by DAG, had a statistically significant effect on implant survival.

### Complications

Postoperative complications were seen in 19/74 patients. The most common was deep wound infection (8), followed by tumor progression (6), aseptic loosening or osteosynthesis failure of reconstruction/osteosynthesis (2), peroneal nerve paralysis (2), and fatal pulmonary embolus (1).

13/19 complications necessitated revision surgery (Tables 2 and 3). 5 patients required subsequent amputation due to prosthetic joint infection (3) or tumor progression (2). In 3 cases, further revision surgery was required to treat a periprosthetic joint infection, either by 2-stage revision in 2 cases, or by debridement, antibiotics, and implant retention (DAIR) in 1 case. In 4 cases treated by osteosynthesis, the fixation failed, which required revision surgery in 2 cases. In the other 2 cases, nonoperative management was advocated due to health deterioration. 3 patients treated with osteosynthesis suffered tumor progression and underwent revision surgery to a tumor prosthesis.

**Table 2.** Complications

Type	Infection	Osteo- synthesis problem	Tumor progression	Permanent nerve palsy	Fatal pulmonary embolism
Tumor prosthesis	8/11	0	1/11	2/11	0
TKA with long cemented stems	0	0	0	0	0
Osteosynthesis with cement	0	2/8	5/8	0	1/8

**Table 3.** Treatment of complications

Type	DAIR	2-stage revision	Amputation	Conversion to prosthesis	Re-osteo- synthesis
Tumor prosthesis	1/7	2/7	4/7	0	0
TKA with long cemented stems	0	0	0	0	0
Osteosynthesis with cement	0	0	1/6	3/6	2/6

6/7 patients with postoperative periprosthetic joint infection (PJI) underwent revision surgery. 3/6 patients had received preoperative radiotherapy. All infections with preoperative radiotherapy occurred in the tumor prosthesis group. There were no infections in the osteosynthesis group (33 patients), despite a comparable incidence of preoperative radiotherapy. None of the 10 patients in TKA group underwent preoperative radiotherapy, and no postoperative infections requiring surgical intervention were seen. The outcome of patients who had received preoperative radiotherapy are summarized in Table 4. The indication for amputation was infection (3) or tumor progression/recurrence (2). 4/5 patients requiring amputation underwent pre- or postoperative radiotherapy.

**Table 4.** Complications and radiotherapy

Type	n	Infection	Osteo- synthesis problem	Tumor progression	Permanent nerve palsy	Fatal pulmonary embolism
Tumor prosthesis (n = 31)						
Preoperative radiotherapy	5	2	0	1	0	0
No preoperative radiotherapy	26	6	0	0	2	0
TKA with long cemented stems (n = 10)						
Preoperative radiotherapy	0	–	–	–	–	–
No preoperative radiotherapy	10	0	0	0	0	0
Osteosynthesis with cement (n = 33)						
Preoperative radiotherapy	7	0	1	1	0	0
No preoperative radiotherapy	26	0	1	1	0	1

## Discussion

The literature describing the outcomes of proximal tibial pathological fractures is limited (Smolle et al. 2019), but what is known is that treatment complications are common for tumors in this location (Mavrogenis et al. 2013). The optimum method for reconstruction lacks consensus.

Reconstruction of the proximal tibia using a tumor endoprosthesis is associated with a high rate of complication and failure (Smolle et al. 2019). Our study demonstrates that this is also true for patients treated with tumor prosthesis for MBD in the proximal tibia. Moreover, the complications were more severe when compared with patients treated with the other surgical methods studied. In particular, the risk of amputation due to infection was highest in patients treated with tumor prosthesis, and infection occurred in the early stages after the operation, consistent with reported findings for proximal tibial replacement following primary malignant bone tumor resection, where infection rates ranged between 6% and 44% (Flint et al. 2006, Myers et al. 2007, Wu et al. 2008, Schwartz et al. 2010, Mavrogenis et al. 2013, Muller et al. 2016). Amputation is the most devastating complication following proximal tibia reconstruction surgery. In our study the rate of amputation after tumor prosthesis infection was 5/8, which is higher than previously reported in primary bone tumor studies with evidently younger patients, where amputation rates due to infection were 5/12 in the study by Tsagozis et al. (2018) and 8/27 in the study by Mavrogenis et al. (2013). The infection rate increases for the lifetime of the prosthesis due to the need for revision and servicing procedures, and can be as high as 87% (Ilyas et al. 2001, Grimer et al. 2002, Plotz et al. 2002, Jeys et al. 2005, Mavrogenis et al. 2011, Mavrogenis et al. 2015). The need for further revision procedures for proximal tibial tumor prosthesis was highlighted by Theil et al., who described the need for further procedures, including the management of infection, in 115/234 patients following a revision procedure (Theil et al. 2019).

The short-term implant survival in the osteosynthesis group was similar to that seen in the endoprosthesis group. Several patients in this group developed complications requiring revision due to infection, failure of fixation, or tumor progression. The need for revision surgery in a plate-osteosynthesis group increases approximately 1 year after primary surgery. Moreover, due to comorbidities, some of these revision procedures were not undertaken. This may represent a selection bias for this method of reconstruction being used in patients in whom survival is considered to be short (less than a year). Due to the intralesional nature of this procedure, adjuvant local or systemic treatment, aimed at reducing the risk of local recurrence or tumor progression, should be considered.

We found the best results, in terms of implant survival or the need for revision surgery, in the cemented long-stemmed TKA group. However, it should be noted that the follow-up time in this patient group was the shortest. As was demonstrated in the other groups, as follow-up increases, the risk of tumor progression increases and thus the need for further surgical intervention. This is reflected by the implant survival rate when studied with competing risk analysis. However, in the short term at least, the incidence of PJI in the long-stem TKA group appears not to present the same challenge as seen in the tumor prosthesis group.

The increased risk of infection when undertaking procedures around the proximal tibia is well established and is at least in part due to the poor soft tissue envelope and the need for extensive dissection to mobilize the proximal tibia. The introduction of gastrocnemius flaps to improve the soft tissue envelope has resulted in a reduction in infections (Myers et al. 2007), but postoperative infection at this site remains higher than for other anatomical locations (Jeys et al. 2005). A gastrocnemius flap was used in all tumor prosthesis reconstructions in our study but, in spite of this, the infection rate remained higher when compared with that seen following resection and reconstruction of a primary malignant tumor of bone. In comparison with the study by Mavrogenis et al. (2013), where complications following proximal tibial replacement were seen in 56 of 225 patients, and the infection rate was 27/225, we found complications in 17/74 patients, with an incidence of infection of 7/74. It is worthy of note that the median age of patients in our study was 64 years compared with 27 years in the study by Mavrogenis et al., which concerned primary malignant bone tumors.

The role of radiotherapy in increasing complications is known (Theil et al. 2019). In our study, radiotherapy prior to tumor prosthesis further increased the risk of infection, despite the addition of a gastrocnemius flap. Radiotherapy leading to PJI often necessitates amputation to eradicate the infection (Mavrogenesis et al. 2011). This must be borne in mind when planning the method of reconstruction to address proximal tibial MBD. Higher age, poor general condition, and suppressed wound healing, for example by antiangiogenetic drugs, further increases the risk of delayed wound healing and subsequent infection (Carroll et al. 2014).

This study does have its limitations. This study is retrospective, with the inherent limitations. However, to our knowledge this is the first study focusing on the surgical management of proximal tibia metastases and, despite the limited numbers, represents the largest cohort to date. The small numbers presented will undoubtedly result in selection bias towards the mechanism of reconstruction. The study does not include patient-related quality of life or functional outcome. Thus a less interventional procedure may carry a better functional outcome but at the risk of tumor progression and the need for later surgical interventions. In addition, it was not possible to accurately assess patient comorbidity status, which may have contributed to the incidence of postoperative complications. Clearly, this must be considered when planning the method of reconstruction in the treatment of MBD in the proximal tibia.

In conclusion, this study highlights the importance of expected patient survival when considering the method of reconstruction of the proximal tibia. For patients with a limited prognosis estimated at 6 months, a plate osteosynthesis with supplemented cement should be considered. For patients with an expected survival in excess of 12 months, a more robust reconstruction should be considered. A cemented long-stem knee replacement provides a durable reconstruction without significant complications, at least in the short term. Tumor prosthesis require prolonged postoperative immobilization due to the need for reconstruction of the extensor mechanism using a gastrocnemius flap, and are associated with a higher incidence of postoperative complications when compared with long-stem cemented knee replacement.

Preoperative radiotherapy may increase the risk of complications, particularly infection, which in the presence of an endoprosthesis may require amputation.
